# Genetic Relationship Between Endometriosis and Melanoma

**DOI:** 10.3389/frph.2021.711123

**Published:** 2021-08-02

**Authors:** Fei Yang, Sally Mortlock, Stuart MacGregor, Mark M. Iles, Maria Teresa Landi, Jianxin Shi, Matthew H. Law, Grant W. Montgomery

**Affiliations:** ^1^The Institute for Molecular Bioscience, The University of Queensland, Brisbane, QLD, Australia; ^2^Statistical Genetics Group, Department of Genetics and Computational Biology, Queensland Institute of Medical Research Berghofer Medical Research Institute, Brisbane, QLD, Australia; ^3^Leeds Institute for Data Analytics, University of Leeds, Leeds, United Kingdom; ^4^Leeds Institute of Medical Research, University of Leeds, Leeds, United Kingdom; ^5^Division of Cancer Epidemiology and Genetics, National Cancer Institute, National Institutes of Health, Bethesda, MD, United States; ^6^School of Biomedical Sciences, Faculty of Health, and Institute of Health and Biomedical Innovation, Queensland University of Technology, Kelvin Grove, QLD, Australia

**Keywords:** endometriosis, melanoma, genetic correlation, genetic risk, mendelian randomization

## Abstract

Epidemiological studies have observed that risk of endometriosis is associated with history of cutaneous melanoma and vice versa. Evidence for shared biological mechanisms between the two traits is limited. The aim of this study was to investigate the genetic correlation and causal relationship between endometriosis and melanoma. Summary statistics from genome-wide association meta-analyses (GWAS) for endometriosis and melanoma were used to estimate the genetic correlation between the traits and Mendelian randomization was used to test for a causal association. When using summary statistics from separate female and male melanoma cohorts we identified a significant positive genetic correlation between melanoma in females and endometriosis (*r*_g_ = 0.144, se = 0.065, *p* = 0.025). However, we find no evidence of a correlation between endometriosis and melanoma in males or a combined melanoma dataset. Endometriosis was not genetically correlated with skin color, red hair, childhood sunburn occasions, ease of skin tanning, or nevus count suggesting that the correlation between endometriosis and melanoma in females is unlikely to be influenced by pigmentary traits. Mendelian Randomization analyses also provided evidence for a relationship between the genetic risk of melanoma in females and endometriosis. Colocalization analysis identified 27 genomic loci jointly associated with the two diseases regions that contain different causal variants influencing each trait independently. This study provides evidence of a small genetic correlation and relationship between the genetic risk of melanoma in females and endometriosis. Genetic risk does not equate to disease occurrence and differences in the pathogenesis and age of onset of both diseases means it is unlikely that occurrence of melanoma causes endometriosis. This study instead provides evidence that having an increased genetic risk for melanoma in females is related to increased risk of endometriosis. Larger GWAS studies with increased power will be required to further investigate these associations.

## Introduction

Endometriosis is a common gynecological disorder, defined as the presence of endometrial like tissue outside of the uterus. The most common locations are in the peritoneal cavity and on the ovary (1). The prevalence of endometriosis is ~10% among women at reproductive age and 19–73% among adolescent females with chronic pelvic pain, significantly impacting their quality of life and work productivity (2). A comorbid relationship between endometriosis and melanoma has been reported (3, 4). Cutaneous melanoma (hereafter melanoma) is a malignant tumor that accounts for ~1% of all skin cancer but represents the most serious type (5), accounting for 75% of skin cancer-related deaths (6). The incidence of melanoma varies greatly between different skin phenotypes and sun exposure with higher rates observed in populations with low pigmentation phototypes living in locations with a high-level of ultraviolet light radiation (7). There are also sex differences in the incidence of melanoma across age groups, the incidence is greater among females than in males before age of 50 (7, 8). A number of epidemiological studies suggest that endometriosis may increase the risk of melanoma (9–12). A retrospective study of 281,937 women from a Scottish national cohort recently found that women with endometriosis (*n* = 17,834) had a higher risk of melanoma compared to women with no evidence of endometriosis (Hazard Ratio = 1.59) (13). However, the estimate was based on a relatively small number of women that had been diagnosed with both endometriosis and melanoma. Epidemiological studies have also indicated that increased endometriosis risk is associated with a history of melanoma, including associations with melanoma-related risk factors such as nevus count, pale skin and red hair, skin sensitivity to sun exposure and the presence of dense freckling (4, 14–19). Epidemiological studies are limited by the necessity for disease diagnosis, differences in age of onset between the diseases and unmeasured confounding factors introducing bias in the results (4). Different sample size and population, choice of control group, as well as case ascertainment through self-report or surgically confirmed disease may also influence the results (20, 21). These limitations are reflected in the conflicting epidemiological evidence of an association between the diseases across studies (3, 4, 17, 18, 22).

Both endometriosis and melanoma are complex diseases with the genetic component playing an important role in disease development. Two large twin studies indicated that ~50% of the variance of susceptibility to endometriosis was attributed to genetic factors (23, 24). Similarly, the heritability of melanoma has been estimated as ~50% (25). Genome-wide association studies (GWAS) have identified 27 risk loci associated with endometriosis, providing strong support for the genetic etiology of this disease (26, 27). The most recent GWAS for melanoma identified 54 risk loci significantly associated with this disease (28). From the perspective of genetic regulation, there are a number of genes located in risk loci that overlap for both melanoma and endometriosis, such as *CDKN2A, CDKN2B, CDK4, TP53, PTEN, KDR*, and *FN1* (27, 29–35). These GWAS datasets provide us with the opportunity to investigate the genetic relationship between these two diseases.

The aim of this study is to investigate the genetic relationship between endometriosis and melanoma, and any confounding effects of pigmentary traits, using data from large GWAS meta-analyses. Evidence of genetic associations between endometriosis and potential risk factors could greatly help us understand endometriosis pathogenesis, identify shared target genes and pathways, and provide useful information for clinicians when considering prevention and diagnostic pathways.

## Materials and Methods

### Data Resources

GWAS summary statistics from the most recent endometriosis (27) and melanoma meta-analyses (28) were utilized in this study. For analytic purposes GWAS datasets were restricted to participants of European ancestry. Summary statistics for eight European cohorts from the endometriosis GWAS meta-analysis in Sapkota et al. (27) were analyzed including 17,405 cases and 191,858 controls. Details of the cohorts are shown in Table 1. GWAS meta-analysis data for melanoma were from Landi et al. (28). This study is the largest melanoma meta-analysis to date with an effective sample size three-times larger than the previous GWAS (28). Excluding self-reported cases, a total of 30,134 cases and 80,415 controls were included in the melanoma meta-analysis; detailed information on the 21 GWAS cohorts included are shown in Table 1. There was limited sample overlap between the melanoma and endometriosis datasets. This overlap is not expected to significantly influence results in excess of a minor proportional bias toward a negative genetic correlation estimate. Endometriosis cases account for <3% of the total melanoma dataset and an even smaller proportion of the effective sample size when considering the ratio of cases to controls.

**Table 1:** Summary of the endometriosis and melanoma case-control cohorts.

**Datasets**	**Number of cases**	**Number of controls**
**Endometriosis**		
QIMRHCS	2,262	2,924
deCODE	1,840	129,016
LEUVEN	998	783
OX	919	5,151
23andMe	4,970	34,561
NHS2-dbGaP	2,238	2,317
WGHS	1,494	14,033
iPSYCH	205	930
**GWAS meta-analysis**	**14,926**	**189,715**
**Melanoma**		
GenoMEL Phase 1	1,075	2,163
GenoMEL Phase 2	1,450	1,128
MDACC	1,924	1,018
AMFS	535	430
Q-MEGA_610k	912	3,777
Q-MEGA_omni	656	538
GSEdinCIDRulcer	4,328	5,780
MELARISK	511	815
WAMHS	1,237	1,977
Essen-Heidelberg	1,189	1,215
Harvard	410	2,920
NCI_CPSII+PLCO+Rose	171	2,684
UK Biobank confirmed	3,499	13,996
MIA_PAH	1,933	2,841
EPIGENE	773	910
QSKIN	1,285	2,493
Greek	1,194	1,279
Italy	1,726	3,142
Spain	3,523	3,400
Michigan	1,198	26,211
BNMS	1,130	2,710
**GWAS meta-analysis**	**30,134**	**81,415**

To explore potential sex differences in the relationship between endometriosis and melanoma, summary statistics from separate male (*n* = 34,157) and female (*n* = 40,123) melanoma cohorts were also included in this study. The separate male and female melanoma and endometriosis datasets were independent with any overlapping samples excluded.

To test whether any relationship between melanoma and endometriosis could be confounded by a genetic correlation with pigmentary traits, GWAS summary statistics, including sex specific GWAS, for pigmentary traits in UK Biobank, generated by the Neale Lab, were downloaded (http://www.nealelab.is/uk-biobank/). Pigmentary traits downloaded included skin color, red hair, childhood sunburn occasions and ease of skin tanning. Similarly, GWAS summary statistics for nevus count generated using 52,806 individuals from 11 cohorts in the USA, United Kingdom (UK), Australia, and the Netherlands, were available to test for correlation with endometriosis (36).

### Genetic Correlation

Using GWAS summary statistics, we investigated the genome-wide genetic correlation (*r*_g_) using LD score regression (LDSC; online methods) (37). LD scores used in the analysis were computed using the 1,000 Genomes European data. We estimated the genetic correlation between endometriosis and melanoma (in males and females combined) and the correlation between endometriosis and melanoma in females/males separately. To test whether genetic correlation estimates for combined melanoma and endometriosis are significantly different to the estimated correlation between endometriosis and melanoma in females and endometriosis and melanoma in males, we applied a *t-*test using the Welch Modified Two-Sample *t*-test included in the BSDA R package.

### Mendelian Randomization

To investigate whether there is a causal relationship between the genetic risk for the two diseases, we conducted bi-directional two-sample Mendelian randomization (MR) using the “TwoSampleMR” R package released with the MR-Base (38). Four MR approaches implemented in the “TwoSampleMR” package were used to estimate the causal effect, each with different assumptions; these include MR-Egger, Weighted median, Inverse variance weighted (IVW) and Simple median. Directional horizontal pleiotropy, where a genetic instrument has an effect on the outcome through another pathway other than the exposure, is a common violation of the assumptions required for valid MR analysis. Therefore, sensitivity tests including a MR-Egger pleiotropy test and the genetic variant heterogeneity test (Rücker's Q statistic) were performed to ensure the causal estimates were not biased (38, 39). A *p* < 0.05 in the pleiotropy test suggested the presence of directional pleiotropy and a Q *p* < 0.05 in the heterogeneity test indicated significant heterogeneity between SNPs. To investigate the effect of including a larger number of SNP instruments for the exposure, we reran the analysis using a GWAS significance threshold to *p* < 5 × 10^−6^.

To further explore the underlying causal relationship between the two diseases, we applied the generalized summary-data based Mendelian randomization (GSMR) method (40). Importantly, GSMR results were less likely to be biased by pleiotropy given that the HEIDI-outlier approach is implemented to remove SNPs with strong potential pleiotropic effects and the causal estimates are highly consistent with MR-Egger slope which is assumed to be robust to pleiotropy. GSMR gains power by considering the sample variation and accounting for the LD among SNP instruments. GSMR was conducted in both directions and SNP instruments from the exposure were selected using a GWAS significance threshold of *p* < 5 × 10^−8^.

### Detecting Genomic Loci Jointly Influencing Both Diseases

We applied a pairwise GWAS (GWAS-PW) (41) to investigate whether the genetic variants in a given region have a causal effect on both endometriosis and melanoma. The whole genome was split into 1,703 approximately independent blocks, and then an extension of the empirical Bayes approach used by Giambartolomei et al. (42) was used to calculate the probability of a region under four models, 1) it contains a genetic variant that affects the first trait only (posterior probability of association, PPA1); 2) it contains a variant that affects the second trait only (PPA2); 3) it contains a variant influencing both traits (PPA3); 4) it contains two different causal variants influencing each trait independently (PPA4). A PPA3>0.9 was used to identify regions with evidence of a shared causal variant. A PPA4 >0.9 was used to identify regions with independent causal variants.

## Results

### Genetic Correlation Between Endometriosis and Melanoma and Its Risk Factors

We assessed the overall genetic correlation between endometriosis and melanoma by conducting a bivariate LDSC analysis. Results showed no evidence of a genetic correlation between melanoma and endometriosis (*r*_g_ = 0.01, se = 0.05, *p* = 0.81) (Table 2). However, after estimating genetic correlations using separate male and female melanoma GWAS datasets, there was evidence of a small significant positive genetic correlation between endometriosis and melanoma in females but not for males (Table 2). The estimated genetic correlation between combined melanoma and endometriosis was not significantly different from that between melanoma in females and endometriosis (*t* = −1.58, df = 97520, *p* = 0.11), nor was there a significant difference between the *r*_*g*_ estimates for endometriosis with melanoma in males and in females (*t* = −1.86, df = 68599, *p* = 0.06). However, considering samples used in the meta-analysis of melanoma in females overlapped with those used in the combined melanoma meta-analysis, the *t*-test results may be biased slightly toward the null. This suggests that although the genetic correlation between melanoma in females and endometriosis is significantly different from zero, we cannot conclude that the *r*_*g*_ estimate in females is significantly different to the combined melanoma estimate. Following correction for multiple testing the *r*_*g*_ estimate of endometriosis and melanoma in females is no longer significant. Larger independent datasets are required to validate the genetic correlation between melanoma and endometriosis.

**Table 2:** Genetic correlation results between endometriosis and melanoma for the combined data and separate analyses for males and females.

	**Combined results**	**Males**	**Females**
*r*_g_	0.013	−0.046	0.144
se	0.053	0.079	0.064
P	0.808	0.563	0.025
SNPs	1,155,952	1,154,197	1,154,204

*r_g_ is genetic correlation; se is standard error; P is p-value; SNPs is the number of SNPs included in the LDSC analysis*.

To investigate whether the relationship between endometriosis and melanoma could be confounded by a genetic correlation with pigmentary traits, we also measured the genetic correlation between endometriosis and skin color, ease of skin tanning, childhood sunburn occasions, red hair and nevi count. Using bivariate LDSC analysis we found no evidence of a significant genetic correlation between any of these traits and endometriosis (Supplementary Table 1).

### Causal Relationship Between Genetic Risk of Melanoma and Endometriosis

**Figure 1:**
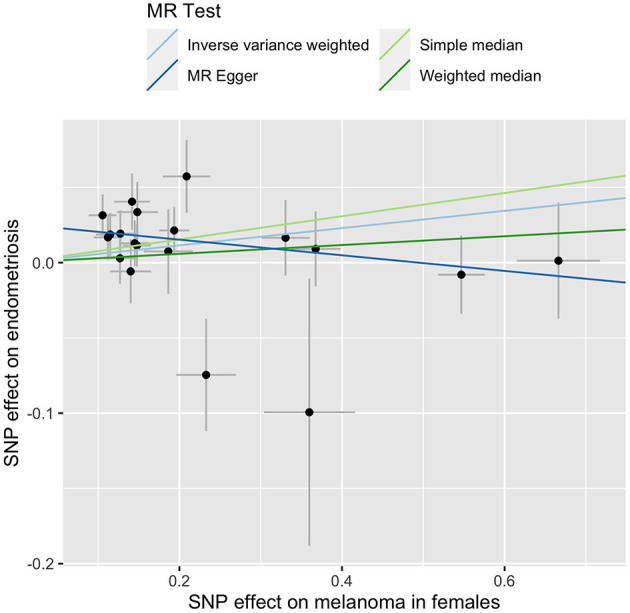
MR-base results for melanoma in females (exposure) and endometriosis (outcome). Different line color represents a different MR test outlined in the key.

We explored the causal relationship between melanoma and endometriosis using MR models in the TwoSampleMR package in R. There was no evidence of a significant causal relationship between the two diseases when the combined melanoma dataset was used (Supplementary Figure 1). However, when conducting the MR analysis using the separate melanoma in females and endometriosis GWAS datasets, we found a small but significant causal relationship when melanoma in females was set as the exposure and endometriosis as the outcome (b_IVW_ = 0.06, se_IVW_ = 0.03, p_IVW_ = 0.03) (Figure 1 and Supplementary Figure 1). Heterogeneity testing using a Cochran's Q test was not significant, showing no evidence of variation in the causal estimates across the SNPs (Table 3). The results of a sensitivity test for directional pleiotropy based on the MR-Egger model was significant with the MR-Egger intercept estimated as 0.03 (se = 0.01, *p* = 0.01), indicating some SNPs may be influencing endometriosis through pleiotropic pathways rather than directly through melanoma. To avoid potential bias the MR analysis was repeated following the removal of all SNPs located in the *MC1R* region, a region strongly associated with red hair phenotype and with large effects on melanoma risk. Results remained consistent before and after removal of the *MC1R* region (b_IVW_ = 0.06, se = 0.03, p_IVW_ = 0.02), showing that the overall causal relationship between genetic risk of melanoma in females and endometriosis was not driven by the *MC1R* region (Table 3). We applied a genome-wide threshold (association *p* < 5 × 10^−8^) and default LD clumping parameter of clump_r2 = 0.001 to select SNP instruments from the female melanoma GWAS dataset resulting in the inclusion of 19 SNPs in the MR analysis. Lowering the GWAS threshold to *p* < 5 × 10^−6^ increased the number of SNP instruments included to 40. Similarly, a small significant causal relationship between the genetic risk of melanoma in females (exposure) and risk of endometriosis (outcome) was identified using the relaxed GWAS threshold based on the MR IVW model (b_IVW_ = 0.04, se_IVW_ = 0.02, p_IVW_ = 0.03) (Table 3). In this case, there was no evidence for heterogeneity or directional pleiotropy. Results from the other three MR-base methods are included in Supplementary Table 2.

**Table 3:** Inverse-Variance Weighted (IVW) Mendelian Randomization (MR) results for melanoma in females (exposure) and endometriosis (outcome).

**GWAS-Threshold**	**MC1R removed**	**Effect size**	**Standard error**	**nSNP**	***P*-value**	**Sensitivity test**
5e-8	YES	0.06	0.03	19	0.02	P(egger) < 0.05, P(he) > 0.05
	NO	0.06	0.03	19	0.03	P(egger) < 0.05, P(he) > 0.05
5e-6	YES	0.05	0.02	39	0.03	P(egger) > 0.05, P(he) > 0.05
	NO	0.04	0.02	40	0.03	P(egger) > 0.05, P(he) > 0.05

*nSNPs is number of SNP instruments included; P-value is the result for IVW MR model; P(egger) is the p-value for the sensitivity test for directional pleiotropy; P(he) is the p-value for the sensitivity test for heterogeneity*.

**Figure 2:**
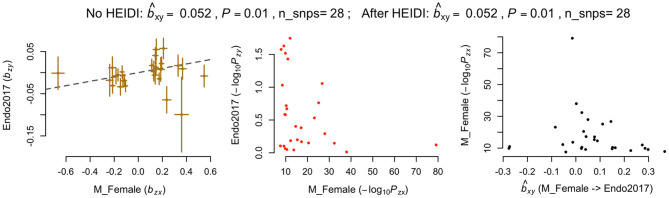
Generalized summary-data based Mendelian randomisation (GSMR) results for melanoma in females and endometriosis. nSNPs, Number of SNP instruments; bxy, estimated effect of the exposure on outcome (bxy = bzy/bzx) where bzy represents the effect of SNP instrument on exposure on the logit scale and bzx represents its effect on outcome free of confounding effect from non-genetic factors.

In addition, we applied another powerful MR analysis approach, GSMR, to further investigate any causal relationships between melanoma and endometriosis. GSMR has an added feature of HEIDI-outlier filtering to remove pleiotropic SNPs so results will not be biased by pleiotropy. GSMR found no evidence of a causal relationship between melanoma and endometriosis using the combined melanoma dataset or the separate male melanoma GWAS datasets. Consistent with results from the two-sample Mendelian randomization method in MR-base, GSMR also identified evidence that the genetic risk of melanoma in females had a significant risk effect on endometriosis (*b* = 0.05, *p* = 0.01) (Figure 2). Compared with the MR-base model, 28 SNP instruments from the female melanoma GWAS were selected for the GSMR analysis using a *p*-value threshold of *p* < 5 × 10^−8^ and default clumping threshold (–clump-r2 0.05). GSMR results for melanoma in females and endometriosis did not change after using HEIDI-outlier filtering, indicating that none of the 28 SNPs had strong pleiotropic effects. After removal of the MC1R region, the causal relationship between the genetic risk of melanoma in females (exposure) and endometriosis remained (*b* = 0.05, *p* = 0.03) (Supplementary Figure 2). Results were consistent between the MR-base and GSMR approach when the MR-base analyses were restricted to the same 28 SNP instruments from the GSMR analysis (Supplementary Table 3). In contrast, neither MR-base or GSMR found significant evidence for a causal relationship between endometriosis as the exposure and melanoma in females as the outcome (Supplementary Table 4). This indicates that the small causal relationship between the genetic risk of melanoma in females (exposure) and endometriosis (outcome) was not biased by the reverse causality model.

We also measured the causal relationship between the combined melanoma and separate male datasets (exposures) and endometriosis (outcome) (Supplementary Figure 1 and Supplementary Table 5). There was no significant effect of melanoma on endometriosis risk using either the combined melanoma or melanoma in males datasets however, in both cases the effect estimates did overlap the 95% confidence interval for the female effect estimate suggesting the effect of the genetic risk of melanoma in females on risk of endometriosis may not be significantly different from the combined and male only estimates.

### Genomic Loci Jointly Associated With Endometriosis and Melanoma

**Figure 3:**
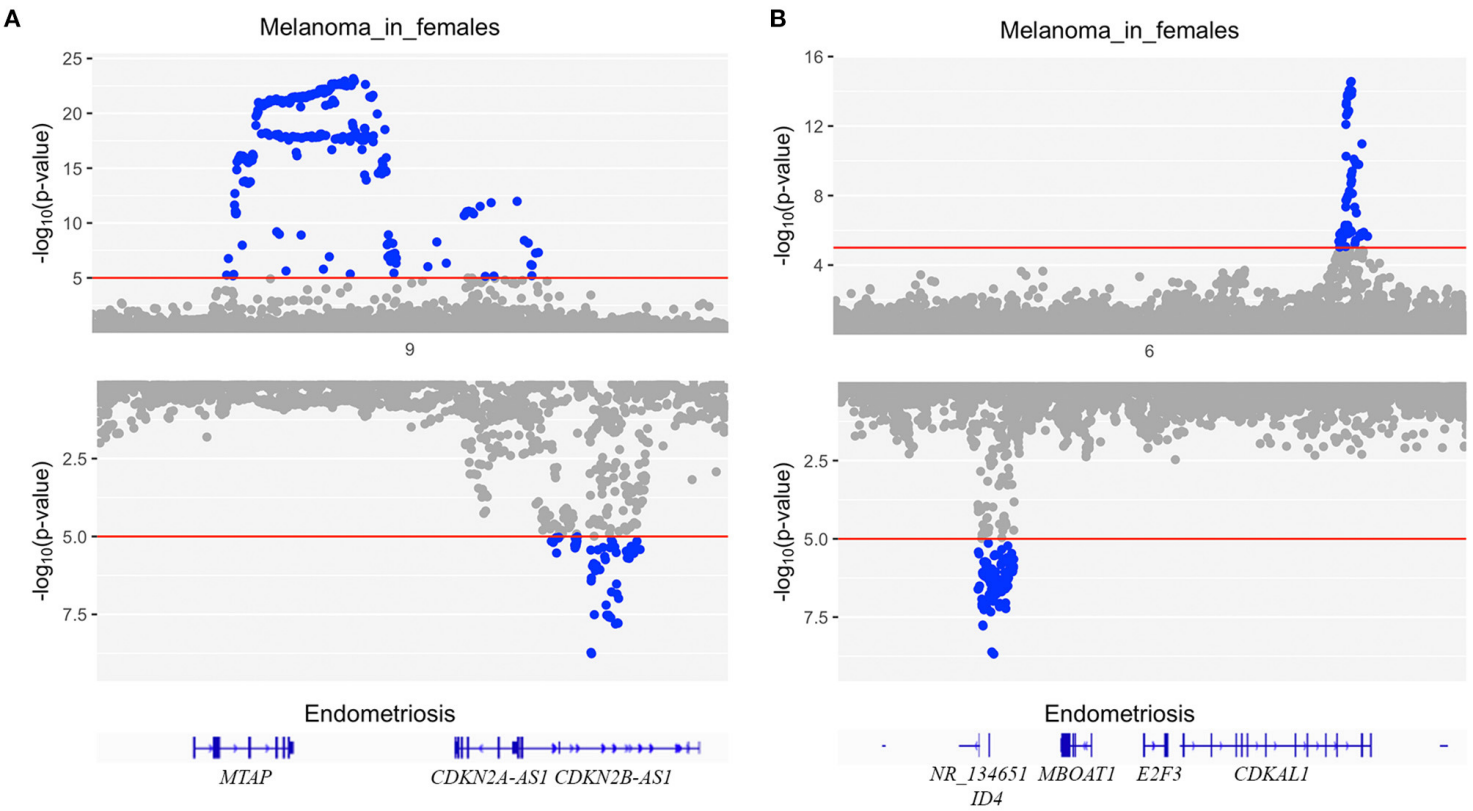
Mirrored Manhattan plots for endometriosis (bottom) and melanoma in females (top) at two regions with the highest probability estimate for independent causal variants in the same risk locus (PPA_4) using GWAS-PW. **(A)** risk locus chr9:20464018-22205246 around *CDKN2A* and **(B)** risk locus chr6:19207758-21683982 around *CDKAL1*.

To identify whether any genomic regions contained causal variants influencing both endometriosis and melanoma in females, we ran GWAS-PW using GWAS results from melanoma in females and endometriosis. Interestingly, there was no evidence of any genomic regions with a shared causal variant for both diseases (PPA_3 < 0.5 for all regions) however, 27 regions had PPA_4 > 0.9 with distinct causal variants influencing endometriosis and melanoma in females independently (Supplementary Table 6). The two regions with the highest probability estimate for independent causal variants in the same risk locus are chr9:20464018-22205246 around *CDKN2A* and chr6:19207758-21683982 around *CDKAL1*. Figure 3 shows a clear distinction between the two signals for each disease.

## Discussion

Epidemiological studies have reported an association between the occurrence of endometriosis and melanoma, two diseases recognized as having unrelated pathologies. This study aimed to assess the genetic relationship between these two diseases leveraging the power of GWAS summary data generated from large cohorts. Using bivariate LDSC analysis we identified a small but significant genetic correlation between endometriosis and melanoma when the melanoma GWAS was restricted to females only. No genetic correlation was observed between endometriosis and the combined melanoma dataset or melanoma in males. The results of the LDSC regression analysis are consistent with the epidemiological studies that, within female cohorts, a history of endometriosis increased the risk of melanoma and vice versa (9, 14, 15). The difference in genetic correlation estimates between the male and female melanoma cohorts may suggest some differences in the genetic architecture for melanoma between females and males. Observational studies also implicated a significant gender divergence in terms of the melanoma incidence rate (7, 43). However, the melanoma meta-GWAS analysis conducted by Landi et al. did not identify any novel sex specific loci when separating the analysis by sex (28). Additional studies need to be conducted to replicate and validate this association when more powerful datasets are available.

In addition to melanoma, epidemiological studies have reported that pigmentary traits and nevus count were also associated with endometriosis risk (4, 14, 15). It is well-known that nevus and pigmentation are risk factors for melanoma and are genetically correlated with the disease (28). In the current study, we found no significant evidence of genetic correlations between pigmentary traits and endometriosis either using the combined or sex specific GWAS datasets. These findings suggest that the genetic correlation between melanoma in females and endometriosis is unlikely to be meditated by genetic regulation of pigmentation or nevus pathways.

Following evidence of a small but significant genetic correlation between melanoma in females and endometriosis, we conducted an MR analysis and provided evidence of causality between genetic risk for melanoma in females (exposure) and endometriosis (outcome). Our findings again support epidemiological observations of an association between risk of endometriosis and risk of melanoma in females however, we find no evidence of causal relationship when using genetic risk estimates from overall melanoma (9, 14, 15). It is important to note that genetic risk is not equivalent to disease occurrence and only explains a proportion of the variance in disease risk. Given the vast differences in disease pathogenesis and age of onset, it is unlikely that melanoma itself causes endometriosis; rather this study provides evidence that having an increased genetic risk for melanoma in females may in fact increase risk of endometriosis. The relationship is more likely to be a result of pleiotropy and/or shared underlying pathways.

Using GWAS-PW we found no evidence for shared causal variants between melanoma in females and endometriosis. This result was not unexpected given the genetic correlation between the diseases was only 0.15, suggesting there are relatively few loci in common and it is likely very large samples sizes would be required to identify these shared loci. Interestingly, there was strong evidence for distinct causal variants influencing the two diseases independently at 27 genomic regions (PPA_4 > 0.9). The two regions with the highest PPA_4 chr9:20464018-22205246 around *CDKN2A* and chr6:19207758-21683982 around *CDKAL1* were both significantly associated with endometriosis and melanoma at a genome-wide threshold but different index SNPs were reported in the different diseases (27, 28), consistent with the current GWAS-PW results. Among the remaining of 25 regions reaching PPA_4 > 0.9, one was previously reported both significantly associated with endometriosis and melanoma, 19 were only reported as genome-wide significant in melanoma, and the remaining five were not genome-wide significant in either endometriosis or melanoma in females (27, 28). The large number of regions containing distinct variants for endometriosis and melanoma may indicate that these two disorders share an underlying genetic architecture which cannot be accurately detected by the current statistical methods and datasets. GWAS-PW is limited by the power of the individual GWAS datasets and the assumption that each trait only has a single causal variant in each region which may not be true in reality (41).

This study provides preliminary genetic evidence of a possible causal relationship between genetic risk of melanoma in females and endometriosis. The biological mechanisms underlying this small causal effect remain unclear. Potential mechanisms include hormonal regulation of cell growth and proliferation and cell cycle regulation. Endometriosis has been characterized as an inflammatory and estrogen dependent disorder (44). Notably, skin itself also functions as an endocrine organ which can possess various kinds of enzymes required for the synthesis of hormones (45). Melanocytes express specific estrogen receptors and estrogen has also been suggested to play a role in the development and progression of melanoma (46–51). Differences in the melanoma survival rate between males and females has been ascribed to the different awareness of risk detection, different lifestyles with men more likely to work outdoors and less likely to apply sunscreen, and to differences in longevity (52–55). However, after these factors were taken into account, the prognostic advantage of women over men still existed, suggesting the female sex hormone may be involved in this protective process (53). Data has shown that sex steroids can influence growth of melanoma cells *in vitro* and that using oral contraceptives may increase risk of melanoma suggesting that melanoma may not be a hormone dependent disease but a hormone sensitive or responsive disease (56). However, despite advances in the area of hormone regulation and melanoma, the exact intracellular pathways connecting estrogen, estrogen receptors and melanoma are still not fully understood (56). Studies have also demonstrated a potential role of cell cycle regulation in both endometriosis and melanoma including the role of increased proliferation and decreased apoptosis in endometriosis progression (57, 58), and risk loci located near known cell regulators, like *CDKN2A*, in both diseases (27, 28). The potential role of hormone and cell cycle dysregulation in the relationship between melanoma and endometriosis requires more in-depth investigation.

Limitations in this study may influence the strength of evidence for an association between endometriosis and melanoma. Firstly, endometriosis is a heterogenous disease likely to collectively describe multiple disease subtypes. The latest GWAS meta-analysis for endometriosis detected differences in the effect size of lead loci between stage I/II and stage III/IV disease and infertility-associated endometriosis cases compared with the overall endometriosis, indicating distinct pathways may be involved in the specific subtypes (26). As such we cannot rule out the possibility of a causal relationship between specific subtypes of endometriosis (exposure) and melanoma (outcome) without validation in larger datasets with more comprehensive phenotyping. Secondly, assumptions of the MR analyses may be violated. One of the IVW MR and GSMR assumptions is that the genetic instruments are not correlated with any confounder in the exposure-outcome association (38, 40). MR-Egger assumed that the genetic effects on the exposure are uncorrelated to the genetic effects on the outcome which is known as the InSIDE assumption (INstrument Strength Independent of Direct Effect) (38). Considering the widely reported polygenicity of complex diseases, it is possible that some of the SNP instruments used in the MR analyses also have genetic effects on a confounding factor biasing the causal magnitude of the exposure on the outcome. This will invalidate both the IVW, MR-Egger and GSMR analyses. Potential confounders may include hormonal related traits such as age at menarche and age at menopause which are both reported to be associated with endometriosis and melanoma (26, 55, 59). Finally, larger, more powerful GWAS datasets would be required to validate these results and achieve a more accurate estimate of genetic correlation and causal effect between melanoma and endometriosis.

In summary, we do not find strong evidence for the genetic correlation between melanoma and endometriosis. However, after restricting the melanoma GWAS meta-analysis to female cohorts, we provide consistent evidence of a small but significant genetic correlation and causal relationship between the genetic risk of melanoma in females (exposure) and endometriosis (outcome) with potential implications in understanding both diseases. We find no evidence that this relationship is driven by pigmentary traits associated with melanoma. Investigation of shared risk loci identified multiple causal variants in these regions affecting endometriosis and melanoma independently. Further studies in larger datasets would be required to confirm these results, uncover specific loci underlying the relationship and rule out effects of pleiotropy, potential confounding factors and ascertainment bias.

## Data Availability Statement

The present study was based on a secondary analysis of GWAS data and all data generated during the study are included in this published article [and its Supplementary Materials]. The endometriosis GWAS data were sourced from the International Endometriosis Genetics Consortium (IEGC); for access to these, contact the consortium directly. Genome-wide summary statistics for the melanoma meta374 analysis have been made publicly available at dbGaP (phs001868.v1.p1), with the exclusion of self-reported data from 23andMe and UK Biobank.

## Ethics Statement

Ethical review and approval was not required for the study on human participants in accordance with the local legislation and institutional requirements. The patients/participants provided their written informed consent to participate in this study.

## Author Contributions

FY, SMo, and GM designed the study with input from the other authors. SMa, MI, MTL, JS, MHL, and GM coordinated data collection, quality control of data, data management, and analysis of the original GWAS datasets. FY, SMo, and MHL ran additional quality control and filtering of GWAS datasets. Data analysis was performed by FY which was interpreted by all authors. FY, SMo, MHL, and GM drafted the report with input from all other authors. The final manuscript has been critically revised and approved by all authors.

## Conflict of Interest

The authors declare that the research was conducted in the absence of any commercial or financial relationships that could be construed as a potential conflict of interest. The handling editor declared a past co-authorship with several of the authors SM and GM.

## Publisher's Note

All claims expressed in this article are solely those of the authors and do not necessarily represent those of their affiliated organizations, or those of the publisher, the editors and the reviewers. Any product that may be evaluated in this article, or claim that may be made by its manufacturer, is not guaranteed or endorsed by the publisher.
