# Supplementary material for: Genetic Relationship Between Endometriosis and Melanoma
Source: Front Reprod Health. 2021 Aug 2;3:711123. doi: 10.3389/frph.2021.711123 (PMC9580819; doi:10.3389/frph.2021.711123)
Supplement: Supplementary file 1 [file Data_Sheet_1.docx]

Supplementary Material

| **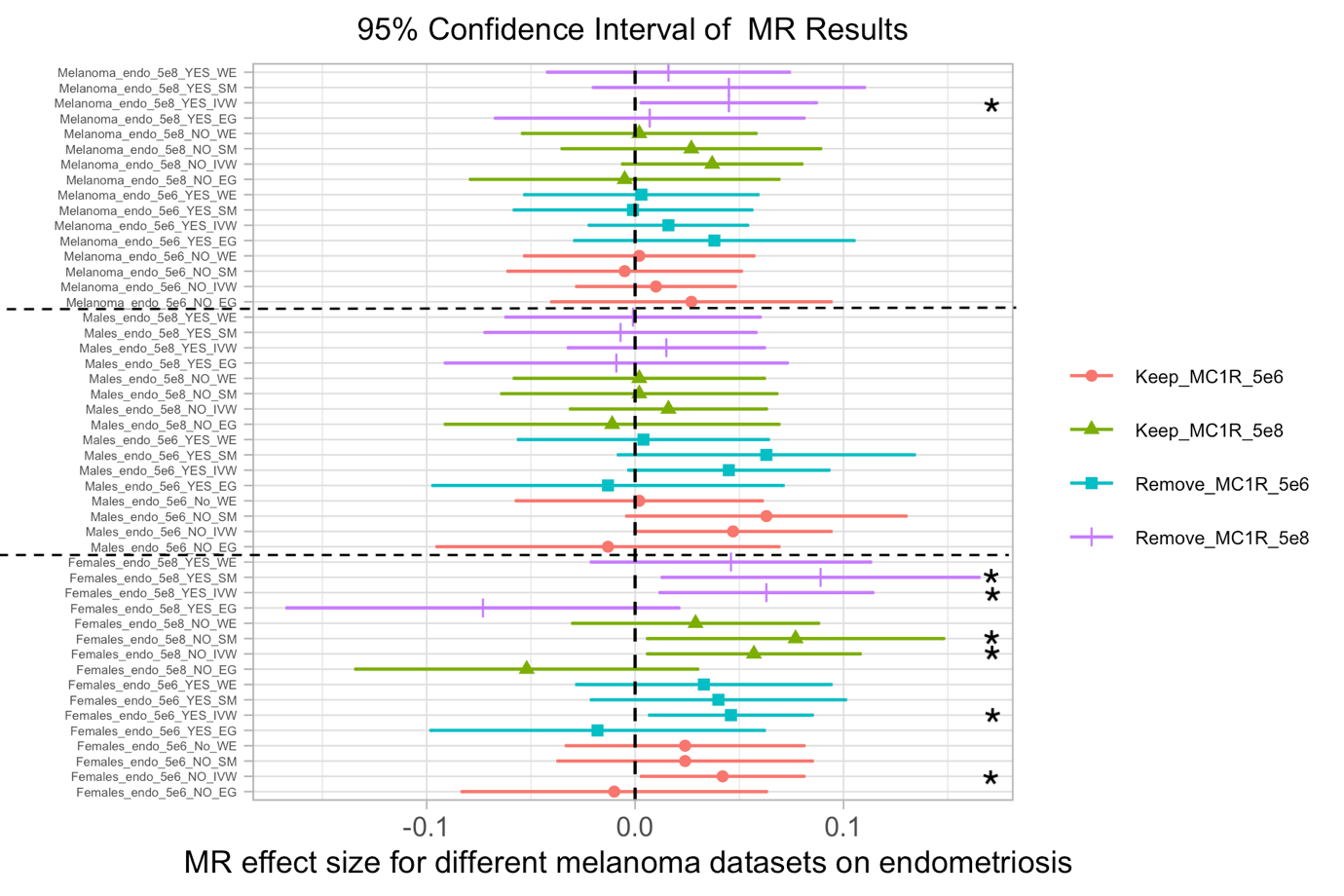** |
| --- |

**Supplementary Figure 1.** Distributions of Mendelian Randomisation (MR) analysis between three different melanoma datasets (exposure) and endometriosis (outcome) using all four MR methods included in MR-base. ***** indicates significant MR results (*P* < 0.05). Different colour represents different GWAS threshold and whether the MC1R region was removed or not. y axis represents each MR method (represented by the capitalized first letter) for each comparison. For example, “Melanoma_endo_5e8_YES_WE” represents the MR analysis for combined melanoma (exposure) and endometriosis(outcome) using Weighted median method, and the GWAS threshold for exposure was set as 5e-8 and the MC1R region was included.


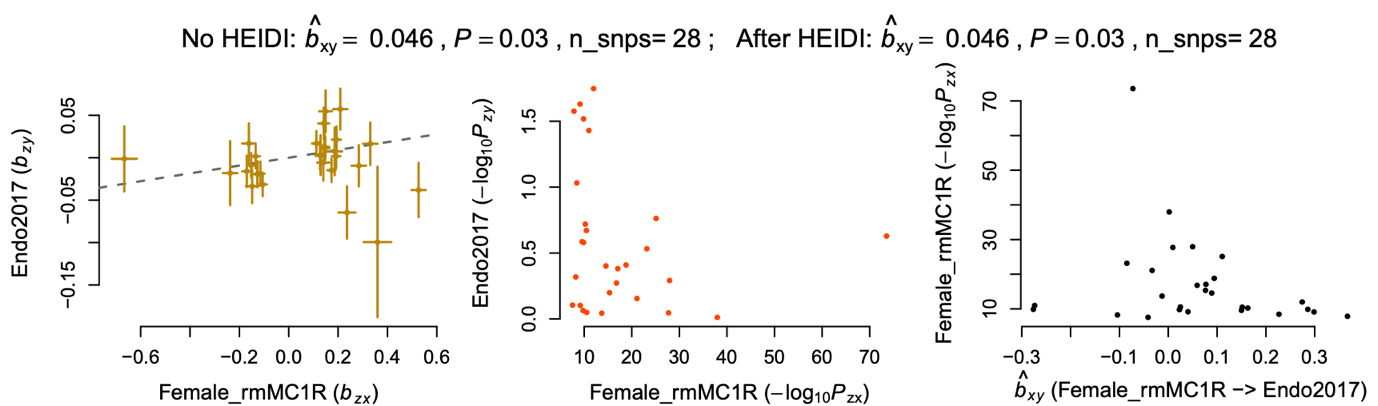


**Supplementary Figure 2.** Results of the generalized summary-data based Mendelian randomisation (GSMR) analysis between melanoma in females (exposure) and endometriosis (outcome) after the removal of the *MC1R* region. nSNPs: Number of SNP instruments; bxy: estimated effect of the exposure on outcome (bxy=bzy/bzx) where bzy represents the effect of SNP instrument on exposure on the logit scale and bzx represents its effect on outcome free of confounding effect from non-genetic factors.

**Supplementary Table 1.** Genetic correlation between endometriosis and pigmentary traits estimated using bivariate LDSC.

| **Trait** | **Dataset** | ***r*_g_** | **se** | **p** | **SNPs** |
| --- | --- | --- | --- | --- | --- |
| **Skin Colour** | Both | 0.020 | 0.035 | 0.569 | 1154268 |
|  | Males | -0.016 | 0.043 | 0.702 | 1154362 |
|  | Females | 0.065 | 0.035 | 0.065 | 1154336 |
| **Red Hair** | Both | 0.012 | 0.047 | 0.800 | 1154206 |
|  | Males | -0.017 | 0.047 | 0.719 | 1154319 |
|  | Females | 0.015 | 0.047 | 0.745 | 1154272 |
| **Childhood Sunburn Occasions** | Both | 0.008 | 0.051 | 0.867 | 1154394 |
|  | Males | -0.026 | 0.057 | 0.653 | 1154400 |
|  | Females | 0.043 | 0.061 | 0.477 | 1154399 |
| **Ease of Skin Tanning** | Both | -0.019 | 0.033 | 0.560 | 1154232 |
|  | Males | -0.011 | 0.042 | 0.800 | 1154356 |
|  | Females | -0.057 | 0.035 | 0.109 | 1154348 |
| **Nevus Count** | Both | 0.006 | 0.077 | 0.935 | 1154232 |
| Note: *r*_g_ is genetic correlation; se is standard error; P is p-value; SNPs is the number of SNPs included in the LDSC analysis. | | | | | |

**Supplementary Table 2.** Results of Mendelian Randomisation (MR) analysis between melanoma in females (exposure) and endometriosis (outcome) using all four MR methods included in MR-base.

| **GWAS-threshold** | **MC1R**  **Removed** | **MR-base Method** | **nsnp** | **b** | **se** | **pval** |
| --- | --- | --- | --- | --- | --- | --- |
| **5e-08** | NO | Inverse variance weighted | 19 | 0.057 | 0.026 | 0.025 |
|  | NO | Simple median | 19 | 0.077 | 0.036 | 0.030 |
|  | NO | Weighted median | 19 | 0.029 | 0.030 | 0.326 |
|  | NO | MR Egger | 19 | -0.052 | 0.042 | 0.232 |
|  | YES | Inverse variance weighted | 19 | 0.063 | 0.026 | 0.018 |
|  | YES | Simple median | 19 | 0.089 | 0.039 | 0.023 |
|  | YES | Weighted median | 19 | 0.046 | 0.034 | 0.176 |
|  | YES | MR Egger | 19 | -0.073 | 0.048 | 0.146 |
| **5e-06** | NO | Inverse variance weighted | 40 | 0.042 | 0.020 | 0.031 |
|  | NO | Simple median | 40 | 0.024 | 0.031 | 0.438 |
|  | NO | Weighted median | 40 | 0.024 | 0.029 | 0.406 |
|  | NO | MR Egger | 40 | -0.010 | 0.037 | 0.795 |
|  | YES | Inverse variance weighted | 39 | 0.046 | 0.020 | 0.025 |
|  | YES | Simple median | 39 | 0.040 | 0.031 | 0.195 |
|  | YES | Weighted median | 39 | 0.033 | 0.031 | 0.296 |
|  | YES | MR Egger | 39 | -0.018 | 0.041 | 0.670 |
| Note: nsnp is number of SNP instruments included; b is the estimated effect of the exposure on outcome; se is the standard error; pval is the p-value. | | | | | | |

**Supplementary Table 3**. Results of Mendelian Randomisation (MR) analysis between melanoma in females (exposure) and endometriosis (outcome) using the same 28 SNP instruments selected by the generalized summary-data based Mendelian randomisation (GSMR).

| **MR-base method** | **nsnp** | **b** | **se** | **pval** |
| --- | --- | --- | --- | --- |
| Inverse variance weighted | 28 | 0.050 | 0.021 | 0.017 |
| Simple median | 28 | 0.046 | 0.029 | 0.112 |
| Weighted median | 28 | 0.025 | 0.027 | 0.365 |
| MR Egger | 28 | -0.048 | 0.042 | 0.271 |

Note: bxy is the estimated effect of the exposure on outcome; se is the standard error; p is the p-value; nsnp is number of SNP instruments included.

**Supplementary Table 4**. Results of Mendelian Randomisation (MR) analysis of endometriosis (exposure) and melanoma in females (outcome) using the GWAS threshold of p-value < 5e-6 for selecting SNP instruments.

| **MR-Method** | **bxy** | **se** | **p** | **nsnp** |
| --- | --- | --- | --- | --- |
| GSMR | 0.006 | 0.031 | 0.841 | 54 |
| Inverse variance weighted | 0.006 | 0.039 | 0.886 | 45 |
| Simple median | -0.046 | 0.049 | 0.344 | 45 |
| Weighted median | -0.041 | 0.051 | 0.421 | 45 |
| MR Egger | 0.036 | 0.116 | 0.756 | 45 |
| Note: bxy is the estimated effect of the exposure on outcome; se is the standard error; p is the p-value; nsnp is number of SNP instruments included. | | | | |

**Supplementary Table 5.** Results of Mendelian Randomisation (MR) analysis between melanoma/melanoma in males (exposures) and endometriosis (outcome) using generalized summary-data based Mendelian randomisation (GSMR).

| **Exposure** | **Outcome** | **HEIDI Test** | **bxy** | **se** | **p** | **nsnp** |
| --- | --- | --- | --- | --- | --- | --- |
| Melanoma | Endometriosis | YES | 0.028 | 0.018 | 0.113 | 80 |
| Melanoma  (MC1R removed) | Endometriosis | YES | 0.030 | 0.018 | 0.087 | 80 |
|  |  |  |  |  |  |  |
| Melanoma_males | Endometriosis | YES | 0.014 | 0.021 | 0.512 | 20 |
| Melanoma_males  (MC1R removed) | Endometriosis | YES | 0.015 | 0.022 | 0.488 | 19 |
| Note: bxy is the estimated effect of the exposure on outcome; se is the standard error; p is the p-value; nsnp is number of SNP instruments included. | | | | | | |

**Supplementary Table 6.** GWAS-PW results of 27 regions containing distinct causal variants for endometriosis and melanoma in females. PPA; posterior probability of association.

| **No. SNPs** | **Chr** | **Start (bp)** | **Stop (bp)** | **PPA_1** | **PPA_2** | **PPA_3** | **PPA_4** |
| --- | --- | --- | --- | --- | --- | --- | --- |
| 4784 | 9 | 20464018 | 22205246 | 6.84E-19 | 1.89E-05 | 3.04E-06 | 1.000 |
| 7041 | 6 | 19207758 | 21683982 | 2.77E-10 | 2.82E-05 | 3.84E-06 | 1.000 |
| 3556 | 20 | 34961245 | 36907686 | 0.0004 | 0.008 | 0.001 | 0.990 |
| 6711 | 7 | 124168509 | 126518757 | 1.69E-07 | 0.025 | 0.004 | 0.971 |
| 2539 | 22 | 39310797 | 40545595 | 0.0056 | 0.011 | 0.002 | 0.962 |
| 4905 | 9 | 107581749 | 109298040 | 0.0000 | 0.035 | 0.004 | 0.961 |
| 3337 | 20 | 32819871 | 34960201 | 0.0000 | 0.041 | 0.005 | 0.954 |
| 4817 | 1 | 224938520 | 226810375 | 0.0012 | 0.030 | 0.004 | 0.954 |
| 5391 | 11 | 87430571 | 89208854 | 0.0000 | 0.040 | 0.008 | 0.952 |
| 3167 | 8 | 21662306 | 22896124 | 0.0008 | 0.036 | 0.005 | 0.950 |
| 3519 | 15 | 27298112 | 29337873 | 0.0001 | 0.043 | 0.006 | 0.949 |
| 2668 | 2 | 201576284 | 202817892 | 0.0009 | 0.028 | 0.016 | 0.947 |
| 4209 | 5 | 33500180 | 35048602 | 0.0000 | 0.049 | 0.007 | 0.945 |
| 3943 | 11 | 89209985 | 90966414 | 0.0002 | 0.044 | 0.009 | 0.944 |
| 7287 | 7 | 16902510 | 19481290 | 0.0001 | 0.041 | 0.014 | 0.944 |
| 6915 | 21 | 41389551 | 43321426 | 0.0000 | 0.047 | 0.011 | 0.942 |
| 4309 | 5 | 982753 | 2131936 | 0.0000 | 0.052 | 0.006 | 0.942 |
| 3620 | 16 | 89041833 | 90155533 | 0.0000 | 0.053 | 0.006 | 0.941 |
| 3728 | 11 | 68006171 | 69516025 | 0.0000 | 0.048 | 0.012 | 0.940 |
| 993 | 11 | 107844498 | 108436952 | 0.0000 | 0.043 | 0.019 | 0.938 |
| 4891 | 16 | 87647436 | 89040532 | 0.0000 | 0.054 | 0.009 | 0.936 |
| 1869 | 20 | 31615242 | 32809249 | 0.0000 | 0.060 | 0.008 | 0.933 |
| 5306 | 16 | 65938609 | 68840588 | 0.0044 | 0.028 | 0.005 | 0.923 |
| 3104 | 16 | 68841409 | 71046522 | 0.0013 | 0.051 | 0.008 | 0.917 |
| 4073 | 20 | 12447777 | 13688842 | 0.0053 | 0.022 | 0.019 | 0.913 |
| 4113 | 22 | 37570269 | 39306269 | 0.0000 | 0.041 | 0.050 | 0.908 |
| 4551 | 20 | 25344231 | 31614788 | 0.0075 | 0.023 | 0.005 | 0.905 |
